# Timing, Surgical Techniques, and Functional Outcomes in Primary Cleft Palate Repair: A Focused Review of Clinical Evidence

**DOI:** 10.7759/cureus.110304

**Published:** 2026-06-05

**Authors:** Geomara N Guerron-Cuenca, Glen A Silva-Rojas, Galo G Farfán-Cano, Kevin J Silva-Rojas

**Affiliations:** 1 School of Medicine, Catholic University of Santiago of Guayaquil, Guayaquil, ECU; 2 Research, FarCan, SilRoj et Al. Research Group, Guayaquil, ECU; 3 Vice-Dean’s Office for Postgraduate Studies and Continuing Education, Rey Juan Carlos University, Mostoles, ESP; 4 School of Medicine, University of Guayaquil, Guayaquil, ECU; 5 School of Health Sciences, Catholic University of Santiago of Guayaquil, Guayaquil, ECU; 6 Research/Translation, FarCan, SilRoj et Al. Research Group, Guayaquil, ECU

**Keywords:** cleft palate, palatoplasty, speech outcomes, surgical timing, velopharyngeal insufficiency

## Abstract

A structured literature review was conducted across PubMed, Scopus, and Web of Science using predefined search terms related to cleft palate, palatoplasty, timing, and velopharyngeal insufficiency (VPI). The aim was to evaluate the impact of surgical timing and operative techniques on functional outcomes following primary cleft palate repair, with a focus on velopharyngeal function. Randomized controlled trials (RCTs) and observational cohort studies reporting speech-related outcomes were included.

Considerable heterogeneity was identified in surgical timing, techniques, and outcome definitions. Most observational studies did not show a statistically significant association between timing and speech outcomes. Anatomical factors, particularly cleft width and subtype, demonstrated a stronger correlation with postoperative outcomes. Current evidence does not support a universally optimal timing for primary palatal repair. Functional outcomes appear to be influenced by multiple factors beyond timing, including surgical technique and patient-specific characteristics. Standardized outcome measures and multicenter studies are needed to improve comparability and guide clinical decision-making.

## Introduction and background

Cleft palate is a common congenital craniofacial anomaly, with an estimated incidence of 1-1.2 per 1,000 live births [1-3]. Surgical repair is essential to restore and preserve key functions such as speech, hearing, and swallowing, which are closely linked to neurolinguistic development [4].

Despite advances in surgical techniques, the optimal timing of primary palatal repair remains controversial. Early intervention, typically performed around six months of age, has been associated with improved early speech development and lower rates of velopharyngeal insufficiency (VPI), reported in approximately 8% of postoperative cases compared to 15% following repairs at 12 months or later [5,6]. However, concerns persist regarding increased perioperative risks and potential adverse outcomes related to immature anatomical structures. Beyond early improvements in speech-related secondary functions, including reduced rates of phonological errors and delayed articulation, there are ongoing concerns about maxillary growth restriction and subsequent midfacial hypoplasia as potential consequences of early repair. Notably, five-year follow-up data have not demonstrated a significant difference in overall speech function between repairs performed at 12 months or later [7-11]. Conversely, delayed repair, often performed at 12 months or later, may reduce surgical complications but has been associated with poorer speech outcomes in some studies.

Large multicenter trials, including the Scandcleft and TOPS studies, as well as observational studies, represent the most robust attempts to establish evidence-based guidelines for primary cleft palate repair. However, their findings are not directly comparable because of substantial methodological heterogeneity, encompassing single-stage and two-stage protocols, divergent surgical techniques such as Furlow palatoplasty, Sommerlad intravelar veloplasty, and variable flap strategies. Combined approaches, such as the Sommerlad-Furlow technique, have been suggested to improve velopharyngeal competence (VPC) and reduce fistula rates. Interpretation is further complicated by anatomical variation in cleft morphology and demographic factors, including differential access to healthcare and the socioeconomic status of patient populations. Functional outcomes reflect this same fragmentation. VPC rates vary considerably, while fistula rates appear paradoxically elevated in early repair groups despite superior speech outcomes. Furthermore, secondary surgery rates range from 8% to 40% across series. Collectively, this heterogeneity precludes the isolation of surgical timing as an independent determinant of outcome and underlies the persistent lack of consensus regarding an optimal and universally applicable repair strategy [2,5,9,11-32].

This review aims to synthesize the current evidence from randomized and observational studies comparing early and conventional timing of primary palatal repair, with a particular emphasis on velopharyngeal function and speech outcomes, while highlighting the most relevant evidence supporting contemporary surgical protocols and techniques. In doing so, it seeks to provide a balanced, realistic, and comprehensive assessment of the current evidence and clinical practice landscape.

## Review

Materials and methods

Study Design and Search Strategy

A structured literature review was conducted in accordance with PRISMA (Preferred Reporting Items for Systematic reviews and Meta-Analyses) 2020 guidelines. The search was performed on January 25, 2026, across three electronic databases: PubMed, Scopus, and Web of Science. The search strategy combined Medical Subject Headings (MeSH) and free-text terms using the following syntax: "cleft palate" AND "palatoplasty" AND "timing" AND "velopharyngeal insufficiency," applied consistently across all databases.

No temporal restrictions were applied, given the limited availability of high-quality evidence addressing the impact of surgical timing on functional outcomes following cleft palate repair. Applying a publication year filter would have further reduced an already limited evidence base. No language restrictions were imposed; studies published in languages other than English or Spanish were accessed using validated web-based translation tools. Database-specific subject area and article-type filters were applied to optimize retrieval relevance. In Scopus, results were limited to the subject area of Medicine and to articles, while in Web of Science, results were restricted to the categories of Surgery, Pediatrics, Dentistry, Oral Surgery, and Medicine. No additional filters were applied in PubMed beyond the open-access restriction, which was uniformly applied across all three databases. The core search string was identical across all platforms. Database-specific adaptations were limited to the subject area and article-type filters described above, with no modifications to the search terms or Boolean operators.

Study Selection and Data Extraction

**Figure 1:**
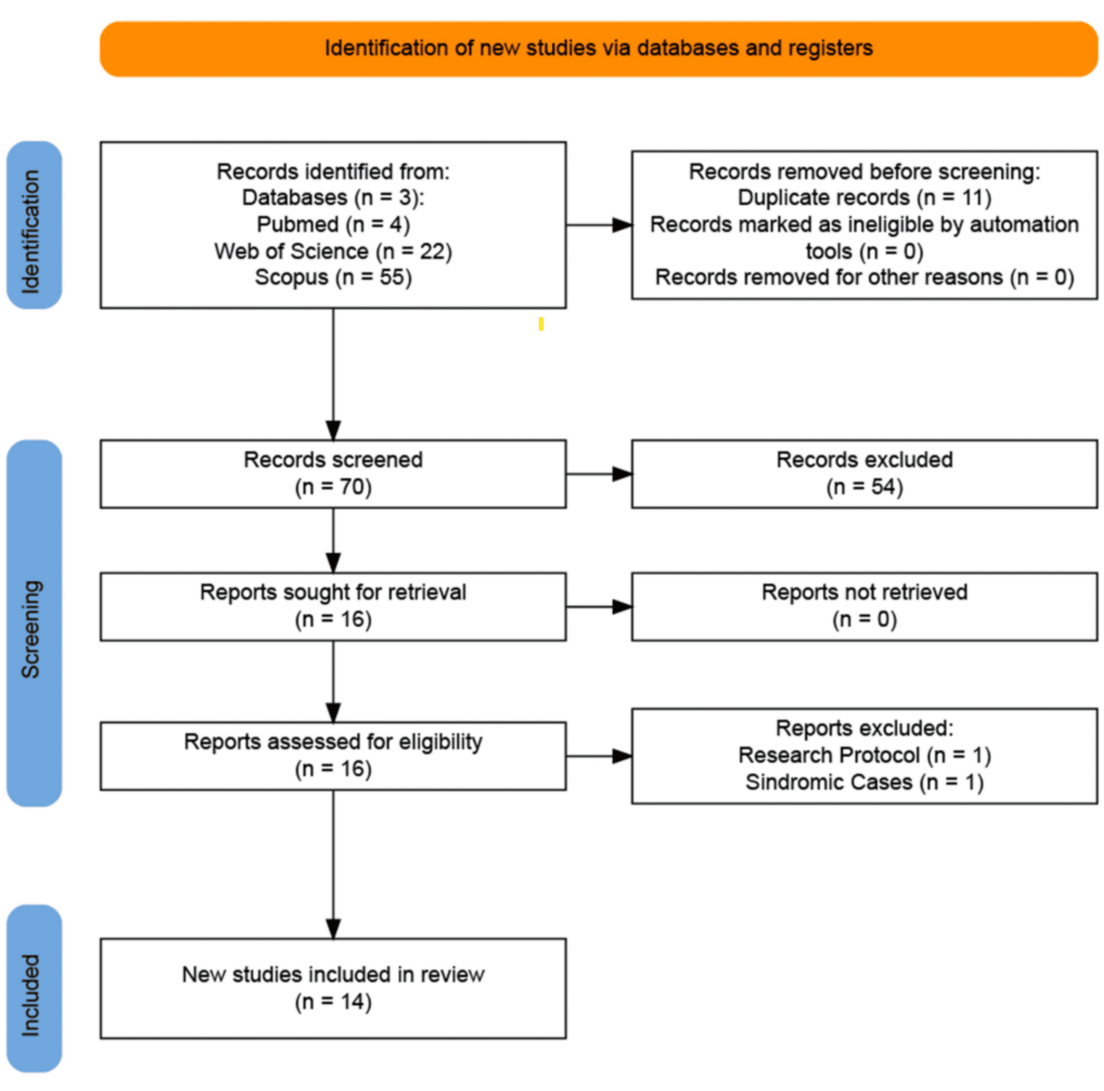
PRISMA flowchart depicting the selection of bibliographic sources The flowchart was constructed using a web-based application [14] PRISMA: Preferred Reporting Items for Systematic Reviews and Meta-Analyses

A total of 81 records were identified (Scopus n=55, Web of Science n=22, PubMed n=4). Duplicate records were automatically removed using Zotero (n=11), leaving 70 records for screening. Following title and abstract screening, 54 records were excluded based on predefined eligibility criteria. Sixteen full-text articles were assessed for eligibility. Two studies were excluded due to methodological limitations, including the inclusion of syndromic patient populations and a non-primary research design (protocol study). A total of 14 studies were included in the final qualitative synthesis (Figure 1).

Study selection was performed independently by two reviewers to reduce selection bias. Discrepancies were resolved through consensus. The Rayyan web-based platform was used to facilitate blinded screening and duplicate detection.

Eligibility Criteria

Studies were included if they met all of the following criteria: a pediatric population (≤18 years) with cleft palate, with or without cleft lip; a clearly defined surgical protocol, including timing of repair (single-stage or staged approaches); and reporting of at least one postoperative functional outcome related to VPC, including VPI, velopharyngeal dysfunction (VPD), speech parameters (e.g., articulation, hypernasality), or the need for secondary surgery. Studies were excluded if they included syndromic or sequence-associated cleft populations; did not report speech-related or velopharyngeal functional outcomes; were review articles, editorials, or other non-primary research; or lacked sufficient methodological detail regarding surgical timing or outcomes.

Outcomes of Interest

The primary outcome of interest was velopharyngeal function, most commonly reported as velopharyngeal insufficiency (VPI) or VPC. Secondary outcomes included speech-related parameters (articulation, hypernasality, phonation) and the need for secondary surgical interventions such as pharyngoplasty.

Quality Assessment

Methodological quality was assessed using the RoB2 tool for randomized controlled trials and the Newcastle-Ottawa Scale (NOS) for observational studies. Studies evaluated with the NOS were categorized according to their aggregate score as low quality (0-3 stars), moderate quality (4-6 stars), or high quality (7-9 stars). Randomized controlled trials (RCTs) were assessed across six domains: randomization process, timing of participant identification, deviations from intended interventions, missing outcome data, outcome measurement, and selection of reported results, and were assigned an overall judgment of low, some concerns, or high risk of bias in accordance with RoB2 criteria.

Data Synthesis

Due to heterogeneity in study design, timing definitions, surgical techniques, and outcome assessment methods, a quantitative meta-analysis was not feasible. Therefore, a qualitative synthesis was performed, focusing on comparative trends between early and conventional timing of primary palatal repair.

Results

A total of two RCTs and 12 observational studies, predominantly retrospective cohort designs, were included. Across the selected studies, substantial variability was observed in surgical techniques, duration of follow-up, and outcome measures used to assess speech performance and velopharyngeal function.

Risk of Bias Assessment

**Figure 2:**
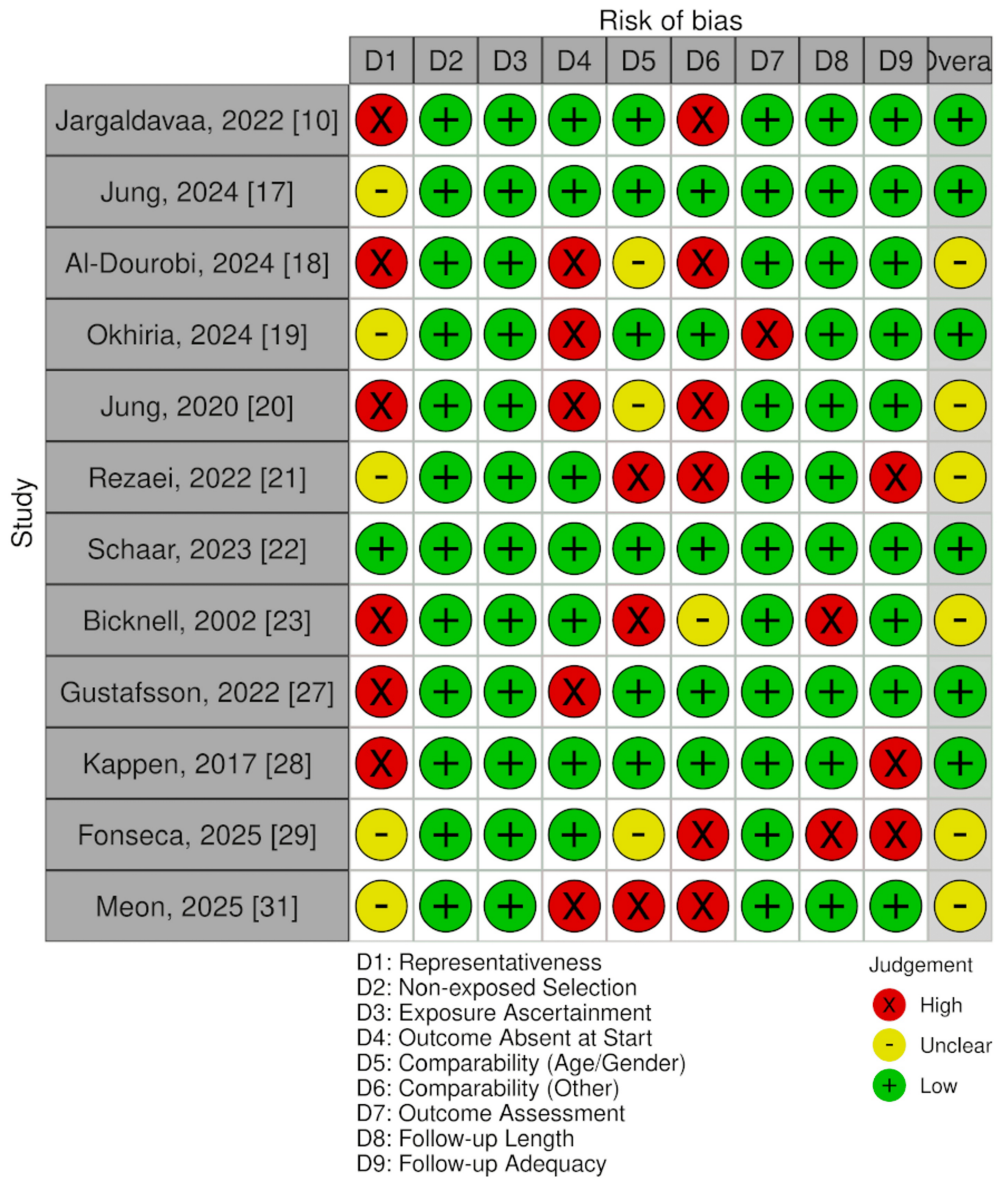
Risk of bias in observational studies: Newcastle-Ottawa Scale tool Newcastle-Ottawa Scale (NOS) assessment tool, constructed using the Robvis visualization application [15] Overa*: overall

The methodological quality of the included observational studies, assessed using the NOS, was generally rated as moderate to high (Figure 2). Most studies demonstrated low risk of bias in domains related to exposure ascertainment and outcome assessment. However, variability was observed in selection-related domains, particularly representativeness and comparability, where several studies showed unclear or high risk of bias.

Follow-up adequacy and duration also presented inconsistencies across studies, potentially affecting the reliability of long-term functional outcomes such as VPI and speech development. Among the RCTs, the RoB2 tool assigned "some concerns" as the overall risk-of-bias rating for both included trials. Both studies demonstrated low risk across the randomization process, timing of participant identification, missing outcome data, outcome measurement, and selection of reported results domains. Some concerns were identified in the deviations from the intended interventions domain for both trials, a finding consistent with the inherent impossibility of blinding participants and operators in surgical research.

Surgical Timing and Protocol Heterogeneity

Considerable heterogeneity was identified in both the definition of surgical timing and the operative strategies employed across studies, as summarized in Table 1. Reported approaches included single-stage repair of the hard and soft palate, as well as two-stage protocols, typically involving early soft palate closure at approximately four months of age, followed by delayed hard palate repair at ≥12 months. In selected cases, particularly in wide cleft defects, hard palate closure was further subdivided into additional stages. Among randomized controlled trials, the TOPS trial compared single-stage repair at six versus 12 months of age, while the Scandcleft trial evaluated mixed protocols, including a two-stage approach (four and 12 months) and a single-stage repair at 12 months. Observational studies demonstrated substantial variability in timing thresholds, most commonly comparing interventions performed before and after 11-13 months, with some studies evaluating delayed repair beyond three years of age.

**Table 1:** Timing of primary palatal repair and velopharyngeal outcomes RCT: randomized controlled trial; VP: velopharyngeal; VPI: velopharyngeal insufficiens; NR: not reported; NOS: Newcastle-Ottawa Scale; RoB2: Risk of Bias 2; VPC: velopharyngeal compentence; VPC-Sum: Velopharyngeal Composite Score; VPD: celopharyngeal disfunction; CMA: compensatory missarticulations; ST: speech therapy

Study	Design	Sample	Timing groups	Primary outcome (VPI/VPC)	Secondary outcomes	Key findings	Secondary surgery	Quality
Jargaldavaa et al., 2022 [10]	Retrospective	335	<18 vs. >18 months	VPI	VP closure	No association (p=0.204)	NR	NOS – High
Gamble et al., 2023 [16]	RCT	552	6 vs. 12 months	VPC-Sum ≥4	Speech	↓ VPI early (8.9% vs 15%, RR=0.59)	Similar	RoB2 – Some concerns
Jung et al., 2024 [17]	Retrospective	374	9–12 vs. 13–18 vs. >18	VPI	Imaging	↑ VPI late (p=0.005)	13.6%	NOS – High
Al-Dourobi et al., 2024 [18]	Retrospective	30	Staged	VPI	Speech	No association	High	NOS – Moderate
Okhiria et al., 2024 [19]	Prospective	62	2-stage	VPC	Anatomy	Anatomy > timing	Variable	NOS – High
Hammarström et al., 2020 [5]	RCT	112	4+12 vs. 12 months	VPC-Sum	Speech	No difference	Similar	RoB2 – Some concerns
Jung et al., 2020 [20]	Retrospective	72	<3y vs. 3–4y vs. >4y	VPI	Speech	Better hypernasality early	8.7%	NOS – Moderate
Rezaei et al., 2022 [21]	Retrospective	180	<13 vs. ≥13 months	VPI	Articulation	Worse CMA late (p=0.021)	NR	NOS – Moderate
Schaar Johansson et al., 2023 [22]	Retrospective	1093	Multiple	VPD surgery	—	↓ secondary surgery early (HR 0.36–0.58)	25.6%	NOS – High
Bicknell et al., 2002 [23]	Retrospective	114	6–18 months	VPI	Pharyngoplasty	No effect	25%	NOS – Moderate
Gustafsson 2022 [27]	Retrospective	290	1 vs. 2 stage	VPI	Surgery	No difference	30.3%	NOS – High
Kappen 2017 [28]	Retrospective	48	2-stage	VPI	Speech	No clear effect	40%	NOS – High
Fonseca 2025 [29]	Retrospective	198	<11 vs. 11–13 vs. >13	VPI	ST referral	No association	NR	NOS – Moderate
Meon 2025 [31]	Retrospective	94	6 vs. 12–18 months	VPI	Phonation	No difference	High success	NOS – Moderate

Velopharyngeal and Speech Outcomes

Findings regarding VPI were inconsistent across studies (Table 1). The TOPS trial reported a lower incidence of VPI in the early repair group compared with the 12-month group (8.9% vs. 15%). In contrast, the Scandcleft trial found no significant differences between protocols (47% vs. 46% in two-stage and single-stage groups, respectively). Most observational studies did not demonstrate a statistically significant association between surgical timing and VPI. However, selected studies reported increased VPI rates following delayed repair, while others suggested improved hypernasality outcomes with earlier intervention. Lower rates of compensatory misarticulations (CMA) were also described in early repair groups. Notably, anatomical variables - particularly cleft width and cleft subtype - were more consistently associated with VPI outcomes than timing alone.

Secondary Surgical Interventions

Rates of secondary surgical intervention for VPI or pharyngoplasty ranged from 8.7% to 40% across studies. The association between the timing of primary repair and the need for secondary surgery remained inconsistent. Schaar et al. reported a reduced hazard ratio (HR) for secondary intervention with earlier repair, whereas the TOPS trial observed slightly higher rates of secondary surgery in the early group compared to the 12-month group (9.7% vs. 5.9%). Speech-related outcomes, including articulation and hypernasality, showed variable results, with some studies favoring earlier repair and others demonstrating no significant differences. Several studies evaluated additional variables, including biological sex, surgical technique, institutional protocols, and adjunctive interventions. These factors were inconsistently reported and demonstrated variable associations with VPI, speech outcomes, and the need for secondary surgical procedures.

Cleft subtype was also identified as a significant modulator of secondary surgery risk. Bilateral cleft lip and palate (BCLP) was significantly associated with increased secondary intervention rates (approximately 50%, p=0.025) [22], while unilateral cleft lip and palate (UCLP) demonstrated lower baseline rates (~14%), increasing up to 40% in delayed or staged reconstructions [31]. Isolated cleft palate repairs performed at 12-13 months showed lower long-term risk of secondary procedures (HR=0.59), whereas multi-stage protocols were associated with higher secondary intervention rates compared to one-stage approaches (HR=2.29) [23]. The main findings of the included studies are summarized in Table 1.

Discussion

Timing for primary palatoplasty remains a highly controversial field. Despite favorable early functional outcomes, particularly in VPC and speech development, reported in observational studies and in the TOPS trial [16-19], an increased incidence of adverse outcomes, including VPI, abnormal speech development, and higher rates of secondary procedures, has also been described. These discrepancies are largely attributed to profound heterogeneity in surgical protocols, encompassing single-stage versus two-stage repair strategies, divergent timing thresholds, and substantially different operative techniques, compounded by variability in outcome measures and patient-related factors such as cleft morphology, access to specialized care, and socioeconomic status. This methodological fragmentation has direct clinical implications; when surgical approach, timing definition, and patient population differ simultaneously across studies, the independent contribution of any single variable to functional outcomes cannot be reliably determined [5,10,21]. The limited availability of high-quality randomized evidence further compounds this challenge, rendering definitive recommendations presently unfeasible.

Currently, no standardized timing for surgical intervention has been established. Early interventions at approximately six months and delayed procedures beyond 18 months remain less commonly adopted and are often considered context-dependent approaches [16,18,22]. Recent evidence suggests that early surgical intervention may support improved early speech development; however, this potential benefit must be balanced against the anatomical and physiological vulnerability of younger patients, which may increase the risk of postoperative complications. This vulnerability is further illustrated by the paradoxically elevated fistula rates observed in early repair cohorts, a finding that is attributable to the restricted tissue dimensions and increased closure tension characteristic of the infant palate, which collectively compromise the biomechanical conditions required for reliable primary wound healing [2,22,23]. Critically, the adverse effects of early palatoplasty are not limited to the perioperative period. Maxillary growth restriction and subsequent midfacial hypoplasia are recognized long-term sequelae that may ultimately necessitate orthognathic correction, including LeFort I advancement, during adolescence.

In the TOPS trial, Gamble et al. reported a significant association between increased VPI incidence when primary palatal repair was performed at 12 months compared with six months at five-year follow-up (Table 1). The primary outcome was rigorously defined as insufficient velopharyngeal function based on the Velopharyngeal Composite Score (VPC-Sum ≥4), a validated ordinal scale derived from three key components: hypernasality, non-oral articulation errors, and VPI-related symptoms. This composite measure, ranging from 0 to 6, was dichotomized to allow clinically interpretable comparisons between groups [16,24,25].

Importantly, although early repair demonstrated improved early functional outcomes, including babbling and hearing-related parameters, no statistically significant differences in long-term speech outcomes were observed at five years when alternative perceptual measures such as VPC-rate were considered. Furthermore, delayed palatal repair beyond 12 months has been associated with higher rates of compensatory misarticulations (70-80%), nasal emissions (~60%), and hypernasality (50-80%), contributing to increased VPI incidence [10,20,21,26]. This highlights the methodological complexity inherent in speech outcome assessment and suggests that results may be influenced by the choice and interpretation of outcome metrics rather than timing alone [16,24,25].

Comparisons between surgical techniques and protocols remain particularly challenging. Commonly used techniques include Furlow double-opposing Z-plasty, two-flap palatoplasty, and the Von Langenbeck procedure. However, few studies have rigorously isolated the independent effect of surgical technique within primary repair [10,17,27]; The TOPS trial standardized the use of the Sommerlad technique across centers, highlighting the importance of controlling surgical variability in comparative research [25].

Reported outcomes vary considerably across techniques. Higher rates of hypernasality and VPI-approaching or exceeding 50% have been described with the two-flap and Von Langenbeck approaches. In contrast, Furlow palatoplasty has demonstrated lower VPI rates (~20%), although its applicability may be limited in wider cleft phenotypes [10,17,18]. These anatomical subtypes, particularly wide cleft geometries, such as unilateral or bilateral complete palatal cleft defects, are themselves associated with increased risk of VPI.

Furthermore, submucous cleft palate (SMCP) represents a significant contributor to VPI, accounting for up to 40% of cases in some cohorts. The Sommerlad technique has been associated with favorable functional outcomes, including VPC rates of 70-80% and Percentage of Consonants Correct (PCC) scores of 80-90% at mid-term follow-up [16,19]; However, these findings must be interpreted cautiously due to variability in surgical expertise, incomplete follow-up, and inconsistent reporting of complications such as infection and fistula formation [23], Additionally, heterogeneity in outcome assessment, particularly subjective and linguistically variable speech evaluation tools, limits cross-study comparability.

Outcomes across surgical protocols remain inconsistent. The Scandcleft trial reported lower VPC rates in one-stage repair (<30%) compared to approximately 43% in two-stage protocols, alongside increased consonant errors in the one-stage group [5]. However, other studies suggest that delayed or staged repair may reduce the need for secondary procedures, highlighting persistent inconsistency in the literature [2,5,17,23,27,28]. These findings indicate that neither timing nor protocol alone sufficiently explains outcome variability. Beyond surgical factors, patient-related and contextual determinants appear to play a critical role. Cleft subtype, nutritional status, and perinatal factors have been associated with postoperative variability [5,16,21,29]. BCLP, hard and soft cleft palate (HSCP), and SMCP are associated with higher rates of secondary surgery [17,21,29].

Van Roey et al. reported that early one-stage hard and soft palatal repair (OSPP) performed at 9-12 months was associated with competent velopharyngeal function in 65.3% and higher PCC scores in 46.7% of UCLP cases at five-year follow-up. In contrast, early or late delayed hard palate closure protocols required subsequent bone grafting between eight and 12 years of age. Speech intelligibility improvement was also more pronounced in the OSPP group. However, at 12- and 22-year follow-up, outcome differences were no longer significant, with VPC and PCC values converging at approximately 60% across groups. Notably, even within the OSPP cohort, speech-correcting surgeries were required in approximately 52% of cases at five years, reinforcing that no single protocol eliminates the need for secondary intervention [30].

Importantly, non-surgical determinants - including access to specialized care, geographic barriers, and socioeconomic factors - substantially influence outcomes. Delayed access to care is associated with increased age at repair and reduced follow-up adherence, while postoperative complications requiring emergency care have been reported in up to 18.2% of cases [29]. Additionally, limited access to speech therapy represents a major confounder, as only a subset of patients receive adequate rehabilitation, directly impacting speech outcomes and compensatory articulation patterns [21].

Secondary procedures do not uniformly result in functional normalization. Outcomes are influenced by multiple factors, including articulation patterns, phonation quality, and timing of primary repair. Although success rates of approximately 80% have been reported in selected early repair cohorts, normalization of phonation often requires additional interventions [19,31]. Moreover, the indication for secondary surgery appears to differ according to timing: early repair is more frequently associated with VPI, whereas delayed repair is more commonly linked to structural complications such as fistula formation [16].

Limitations

This review is limited by the predominance of observational studies and the small number of RCTs, which restricts the strength of causal inference. Additionally, inherent methodological constraints in surgical research, particularly the difficulty of implementing blinding and strict randomization, introduce potential performance and detection biases. The risk of bias assessment revealed variability in study quality, particularly in the selection and comparability domains, which may have influenced reported outcomes. Heterogeneity in surgical techniques, timing definitions, and outcome assessment methods further limits comparability across studies.

Finally, inconsistencies in follow-up duration and outcome reporting, especially for long-term speech development, may affect the reliability of conclusions regarding functional outcomes. The application of an open-access filter across all three databases represents an additional constraint, as relevant subscription-based literature may not have been captured by the search strategy. Studies accessed in languages other than English or Spanish were translated using validated web-based tools, which may have introduced some imprecision in interpreting reported outcomes and methodological details.

## Conclusions

Early surgical intervention may be associated with improved functional outcomes; however, variability among studies and the presence of potential biases continue to prevent firm conclusions. The lack of standardized reporting, particularly regarding cleft subtype, surgical technique, and postoperative complications such as infections, complicates efforts to define an optimal strategy in terms of timing, protocol, or approach. Advances in minimal invasive surgical strategies, such as the Sommerlad technique, represent an important step forward in cleft palate repair. Comparisons between one-stage and two-stage protocols provide valuable insights into outcome variability and procedural success. Although early one-stage repair appears promising, as suggested in the TOPS trial, current evidence remains insufficient to support its universal adoption.

Existing evidence suggests that surgical timing between 9-10 months and up to 18 months may represent a reasonable window for intervention; nonetheless, outcomes are influenced by individual patient factors and the broader surgical context. Variables such as socioeconomic status, caregiver education, and patient medical history have been suggested as potential contributors to surgical success and should be systematically evaluated. Larger multicenter studies with standardized methodologies are required to clarify the relative superiority of surgical techniques, timing, and protocols. The development and universal adoption of a unified clinical assessment tool for VPC and speech outcomes is critically needed, as the current reliance on heterogeneous, subjective, and linguistically variable evaluation instruments represents a major barrier to cross-study comparability and evidence synthesis. Particular attention should be given to secondary procedure rates, as repeated interventions may negatively impact patient well-being and social development.
